# Galectin-10 Is Released in the Nasal Lavage Fluid of Patients with Aspirin-Sensitive Respiratory Disease

**DOI:** 10.1100/2012/474020

**Published:** 2012-04-30

**Authors:** Ma. Cristina Negrete-Garcia, Carla Yoneli Jiménez-Torres, Noe Alvarado-Vásquez, A. Rosalía Montes-Vizuet, J. R. Velázquez-Rodriguez, M. Carmen Jimenez-Martinez, Luis Manuel Teran-Juárez

**Affiliations:** ^1^Department of Immunogenetics and Allergy, Instituto Nacional de Enfermedades Respiratorias, Calzada Tlalpan 4502, 14080 Mexico, DF, Mexico; ^2^Department of Biochemistry, Instituto Nacional de Enfermedades Respiratorias, Calzada Tlalpan 4502, 14080 Mexico, DF, Mexico; ^3^Department of Immunology, Research Unit, Institute of Ophthalmology, Foundation Conde de Valenciana, 06800 Mexico, DF, Mexico

## Abstract

The aim of this work was to determine the presence of galectin-10 in nasal lavage fluid (NLF) of patients with aspirin-sensitive respiratory disease (ASRD) before and after challenge with L-ASA (aspirin) by ELISA. Fifteen ASRD patients, ten aspirin-tolerant asthmatics (ATA), and fifteen healthy controls (HC) were studied. The baseline presence of Galectin-10 in PBMC was determined using real time PCR. Galectin-10 was evaluated in tissue of nasal polyps by western blot. Our results showed a lower expression in PBMC of ASRD patients than in ATA and healthy controls. However, a higher concentration of galectin-10 in NLF was found in ASRD patients before and after L-ASA challenge; western blot confirmed a high expression of galectin-10 in tissue from nasal polyps obtained from ASRD patients. Our results suggest a probable role of galectin-10 in the inflammatory response observed in ASRD patients; however, confirmatory studies are needed.

## 1. Introduction

Aspirin-sensitive respiratory disease (ASRD) is a clinical syndrome characterized by a combination of nasal polyposis, chronic hypertrophic eosinophilic sinusitis, asthma and intolerance to aspirin or other nonsteroidal anti-inflammatory drugs (NSAIDs). Ingestion of aspirin (L-ASA) and of most NSAIDs results in upper and/or lower respiratory reactions, including rhinitis, conjunctivitis, laryngospasm and bronchospasm [1]. The earliest symptoms are usually rhinorrhea with nasal congestion and progression to the lower respiratory tract with asthma and finally nasal polyposis [2]. In a study performed in the USA, the average age of onset was reported as 34 years, and it may be acquired between youth and mature age, with no ethnic predilection and rare familial associations. ASRD is more commonly reported in women than in men (57% versus 43%) [3]. 

Galectins are a growing family of *β* galactoside-binding animal lectins. At least 12 galectins are present in humans [4]. It has been proposed that some galectins are secreted by an unorthodox mechanism to exert their extracellular function [5]. The wide distribution of galectins, with respect to species and tissues, suggests that they exert many different functions. The two most extensively studied galectins are galectin-1 and galectin-3 [6], but little information exists about galectin-10. It is well known that galectin-10 has been localized only in eosinophils and basophils; [7] however, a recent finding shows that galectin-10 is constitutively expressed in CD4+CD25+ regulatory T-cells in addition to playing a role for regulatory T cell function [8].

Currently, the knowledge about the presence of some galectins in epithelium, endothelium, and activiated tissue macrophages, cells that are involved in inflammation, it has led to many researchers to study the role of galectins in inflammatory processes [9]. Taking into account that the ASRD is a clinical syndrome associated with severe and chronic inflammation in both upper and lower airways and a recent finding [10], where the authors reported an overexpression of galectin-10 mRNA in peripheral blood of aspirin-induced asthma, we thought it was interesting to investigate whether galectin-10 was released in the nasal lavage fluid of a group of patients with ASRD before and after the challenge with the triggering agent (L-ASA) and to analyze its probable participation in the associated inflammatory process in ASRD.

## 2. Material and Methods

### 2.1. Subjects

Forty subjects from the Department of Immunogenetics and Allergy of our Institute, participated in this study, all of them were nonsmokers (Table 1). Their atopic status was investigated by skin-prick testing with different allergens including DPT, cat and dog dander, cocroach and trees-like *Ligustrum vulgare or Alnus glutinosa*. Neither oral corticosteroids nor antileukotrienes were given to patients 4 weeks before the study and *β*2-agonists were withdrawn 48 Ih before the study. Aspirin (L-ASA)-sensitivity was established by clinical history and a nasal L-ASA challenge [11]. Before the challenge, all patients were examined with anterior rhinoscopy to evaluate the presence of nasal polyps. Five of the 15 L-ASA sensitive patients had small nasal polyps (Lund-Mackay score = 1). Patients with nasal polyps grades 2 and 3 did not participate in the study, because they could not undergo reliable rhinomanometry measurements [12]. This study was approved by the Institutional Ethics Committee and all patients gave their signed informed consent. 

### 2.2. Study Design

Subjects were divided into three groups: (1) 15 L-ASA-sensitive asthmatics (ASRD), (2) 10 aspirin-tolerant asthmatics (ATA), and (3) 15 healthy controls (HC). The ASRD group had been diagnosed previously by both clinical history and a positive response to a nasal L-ASA challenge (from 25 mg to 100 mg, according to the clinical response). A baseline nasal lavage and a peripheral blood sample were carried out in all subjects during the screening visit. Subsequently, nasal lavage fluid (NLF) was obtained pre- and postnasal challenge with L-ASA [13], which was used for the detection of Galectin-10 by ELISA.

### 2.3. Nasal L-ASA Challenge

A nasal L-ASA challenge was performed using a modified protocol described by Casadevall et al. [11]. The nasal challenge was performed with a total dose of 25 mg of L-ASA (Aspisol, Bayer Germany), 12.5 mg L-ASA was instilled in each nostril and on each inferior turbinate. The nasal response was evaluated by rhinomanometry and clinical response by scoring the subjective nasal symptoms using a visual analogue scale ranging from 0 (no symptoms) to 30 (severe symptoms) as described previously [14]. The clinical symptoms score included rhinorrhea, nasal blockage, itching of the nose, and palate and/or throat and sneezing. The positive nasal reaction was defined as the appearance of nasal symptoms such as rhinorrhea, nasal congestion, sneezing, and a 25% decrease of the total nasal flow value, as compared with the baseline measurement or a 40% bilateral decline of inspiratory nasal flow, as compared with the baseline value assessed by rhinomanometry. Subjects with baseline nasal inspiratory flow of 250 mL/s did not participate in the study. The bronchial response was also evaluated by performing spirometry.

Nasal lavage fluid (NLF), blood, and tissue specimens from HC, ATA, and ASRD subjects were collected under rules approved by the institutional Bioethics Committee.

### 2.4. mRNA Expression by Quantitative Real-Time RT-PCR

RNA was extracted from 8 × 10^6^ PBMC using TRIZOL (Gibco BRL, Gaithersburg, MD) according to manufacturer's instructions. RNA was reverse transcribed into cDNA using the ThermoScript RT-PCR system (Invitrogen, Carlsbad, CA, USA). Galectin-10 mRNA levels were quantified by real-time RT-PCR (qRT-PCR) using TaqMan Gene Expression Assay (Applied Biosystems, Tokio, Japan) on a StepOnePlus Real-Time PCR System (Applied Biosystems) according to manufacturer's instructions. The relative quantification method [15] was used to measure the amounts of Galecin-10, normalized to 18 s rRNA as endogenous control. The results were analyzed with the software ABI PRISM 7700 Sequence Detection System.

### 2.5. Double Antibody Solid Phase Immunoassay (ELISA)

Measurement of galectin-10 was performed using a double antibody solid phase immunoassay (ELISA) developed in our laboratory. A human eosinophil cellular protein extract from a patient's blood with eosinophilia was used as positive control a human. Briefly, a monoclonal antibody to galectin-10 (Abcam, Cambridge UK) was incubated overnight at 4°C in an immunosorbent plate (R&D Systems, Minneapolis MN, USA). The plate was blocked with BSA (1%, 2 h, RT), and 100 *μ*L of nasal lavage fluid (NLF) or positive control was added, each sample was evaluated in triplicate and incubated overnight at 4°C. Later, the plate was washed four times with phosphate-buffer saline (PBS) containing 0.05% Tween 20 and then, polyclonal goat antihuman galectin-10 antibody 1 : 100 (R&D Systems) was added to each well (2 h). After washing with PBS-Tween 20, we added a conjugate of donkey anti-goat biotinylated 1 : 300 (Sigma St. Louis, MO, USA) (2 h, RT). To evidence the reaction, 100 *μ*L/well of ExtrAvidin-Peroxidase 1 : 500 (Sigma) was added for 30 minutes. The plate was washed again and the immunoenzymatic reaction was revealed with tetramethylbenzidine (TMB) substrate (Sigma-Aldrich St. Louis, MO, USA). The absorbance was measured at 450 nm in an ELISA plate reader (Benchmark, Bio-Rad, Hercules, CA,USA).

### 2.6. Western Blot

To evaluate the presence of galectin-10 in tissue samples, we performed a western blott with the protein extract of polyps obtained from ASRD and ATA patients. Tissue of nasal mucosa without inflammatory process was used as negative control. Nasal tissue extracts were prepared with cold lysis buffer (2 M Tris, pH 7.5, 0.15 M NaCl, 0.05 M NaF). Each sample was centrifuged at 13,000 rpm (Centrifuge Hermle Z232K, Hermle LaborTechnik, GmbH, Germany) for one minute at 4°C. The supernatant was separated and the total protein concentration was determined with the Pierce BCA Protein assay kit (Thermo Scientific). Proteins (previously adjusted to 30 *μ*g) were separated on 12% SDS Laemmli gels [16]. After electrophoresis, the proteins were transferred onto a polyvinyl difluoride membrane (Bio-Rad), the membrane was incubated with 3% BSA for 2 hours and then incubated with a monoclonal antibody to galectin-10 (Abcam, Cambridge, UK, 1 : 200) overnight at 4°C. Immunodetection was performed incubating with a monoclonal anti-mouse biotinylated antibody and the Streptavidin-Peroxidase conjugate (1 : 1000) (Sigma) for 30 minutes; afterwards, the membrane was washed and incubated with DAB (Vector Laboratories, Burlingame, CA, USA).

### 2.7. Statistical Analysis

Statistical analysis was performed using Mann Whitney test, (GraphPad Prism 5, from GraphPad software, Inc. CA, USA). *P* ≤ 0.05 was considered statistically significant. A post-hoc analysis of the sample size using G power (version 3.0.10) showed that 15 ASRD and 10 ATA subjects would have sufficient power (80%) to test our hypothesis.

## 3. Results

### 3.1. Clinical Findings

Intranasal challenge with L-ASA-induced rhinorrhea, nasal congestion, and sneezing symptoms lasted up to 2 h in all ASA-sensitive patients. Following L-ASA challenge, a significant decline in the mean nasal inspiratory flow in ASA-sensitive patients was observed. Absence of clinical response to L-ASA was observed in both aspirin-tolerant asthmatics (ATA) and healthy controls (HC).

### 3.2. Gene-Expression of Galectin-10

**Figure 1:**
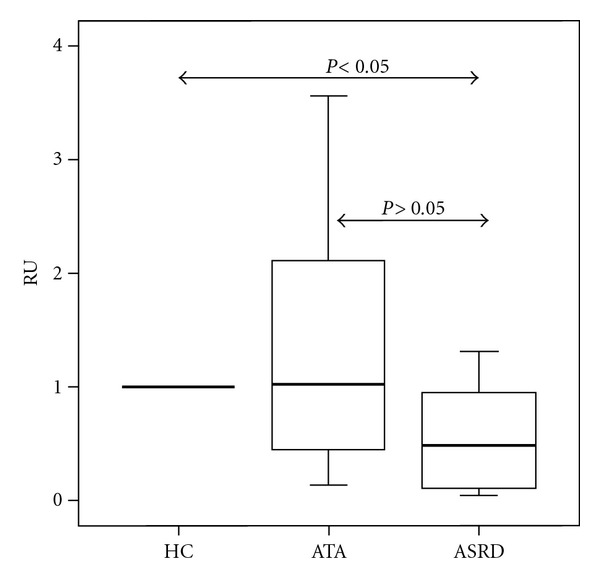
Relative quantification (RU:Relative Units) of galectin-10 gene expression in PBMC with respect to healthy controls (HC) in patients with aspirin sensitive respiratory disease (ASRD) and aspirin-tolerant asthma (ATA).

We analyzed the galectin-10 gene expression in PBMC by real time PCR (Figure 1). The values were determined with respect to the healthy controls. A lower expression of galectin-10 gene was observed in ASRD with respect to ATA patients (0.681 ± 0.733 versus 1.262 ± 1.107). 

### 3.3. Galectin-10 in Nasal Lavage Fluid

**Figure 2:**
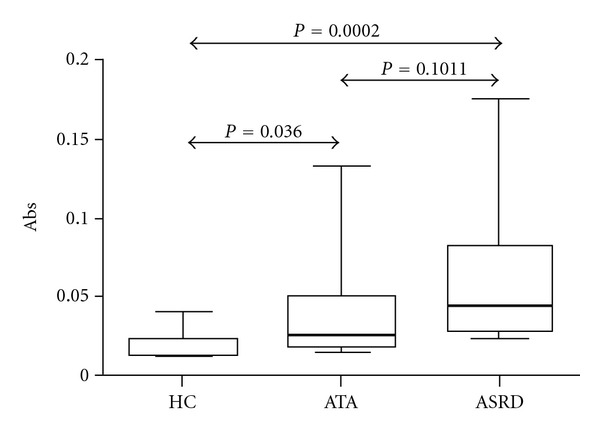
Determination of galectin-10 by ELISA at baseline in nasal lavage fluid of aspirin sensitive respiratory disease patients (ASRD), aspirin-tolerant asthmatics (ATA) and healthy controls (HC).

**Figure 3:**
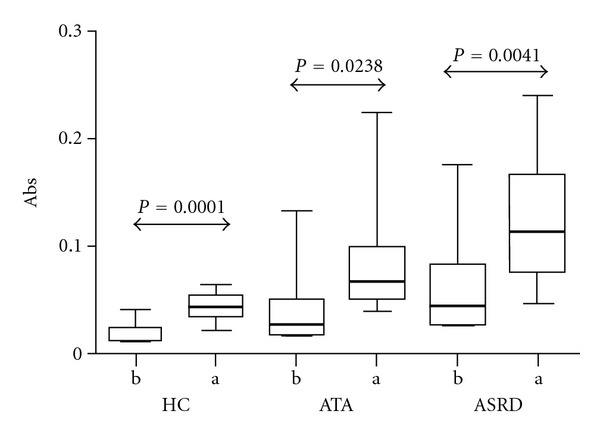
Comparison of galectin-10 levels before (b) and after (a) nasal challenge with L-ASA in nasal fluid lavage of aspirin-sensitive respiratory disease patients (ASRD), aspirin-tolerant asthmatics (ATA) and healthy controls (HC).

A higher baseline concentration of galectin-10 was observed in the NLF from ASRD and ATA patients with respect to the HC (0.045 ± 0.046, 0.034 ± 0.048 versus 0.015 ± 0.011, resp.) (Figure 2). Interestingly, when we compared the pre- and postnasal L-ASA-challenge galectin-10 levels, significant differences in the concentration of galectin-10 were observed (Figure 3) (ASRD: 0.045 ± 0.046 versus 0.114 ± 0.064, ATA: 0.027 ± 0.039 versus 0.068 ± 0.059 and HC: 0.015 ± 0.010 versus 0.044 ± 0.014); these differences became more evident when comparing between ASRD and ATA patients (*P* < 0.0041 versus *P* < 0.05). 

### 3.4. Detection of Galecin-10 in Nasal Tissue by Immunoblot

**Figure 4:**
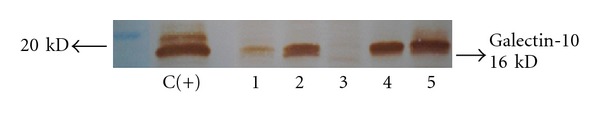
Western blot analysis of galectin-10 in supernatant of lysed nasal polyp from an aspirin-tolerant asthma polyp (lane 1), aspirin sensitive respiratory disease nasal polyps (lane 2, 4, and 5) and nasal mucosa as a tissue control without inflammatory process (lane 3). C(+) protein extract of eosinophils. (First lane, molecular weight markers).

Galectin-10 expression in nasal tissue was investigated by immunoblot using tissue from nasal polyps of ASRD and ATA patients and comparing their values versus healthy nasal mucosa tissue. Protein extract of eosinophils was used as positive control (Figure 4). Nasal polyps from the three different patients with ASRD and ATA were evaluated. Results show a higher expression of galectin-10 in patients with ASRD when compared with ATA patients and healthy nasal tissue. In healthy mucosa tissue, a lower galectin-10 expression was observed. This higher expression of galectin-10 in patients with ASRD diagnosis may be probably a consequence of the more aggressive inflammatory process observed in this disease. 

## 4. Discussion

This is the first study that shows the release of galectin-10 in nasal fluid lavage (NLF) of aspirin-sensitive respiratory disease (ASRD) patients before and after nasally challenged with L-ASA. The galectins family include 15 members [4, 17], which are *β*-galactosides-binding animal lectins [18]. Galectins have been associated with the innate and adaptive immune function [19, 20], as well as with cancer, immunity, and inflammatory responses [20, 21]. Galectin-10 or Charcot Leyden crystal protein (CLC) has been previously related with the inflammatory process [6] and, recently also, it has been found in patients with allergic rhinitis [22]. However, its function in the cell is still only partially understood. In this work, the expression of galectin-10 by real time PCR was determined in PBMC obtained from patients with ASRD. Recently, Devouassoux and cols. (2008) [10] reported an overexpression of mRNA galectin-10 in whole blood samples from patients with ASRD in absence of challenge with L-aspirin, and they suggested the importance of galectin-10 in the accurate diagnosis of aspirin-induced asthma. Our results, however, showed a minor baseline expression of galectin-10 in PBMC from ASRD patients. It is probable that the observed differences in the expression of galectin-10 could be an effect of the purification process of RNA [23], or due to individual differences in the galectin-10 gene-expression patterns as a product of the evaluated patient's age [24]. Additionally, it is important to highlight that the diagnosis of ASRD in the group of patients sutudied by Devouassoux was not made with the aspirin challenge test, regarded as decisive for the correct diagnosis of ASRD [25].

In this investigation, we also analyzed the presence of galectin-10 in healthy nasal tissue and nasal polyps extracts from ASRD and ATA patients by western blot. Our results showed a higher expression of galectin-10 in nasal polyps of patients with ASRD with respect to the ATA or healthy nasal tissue subjects. The presence of galectin-10 was confirmed using protein eosinophil extract as a positive control. Previously, the galectin-10 protein had been found in diverse tissues, body fluids, or cells involved with the inflammatory response, like eosinophils and basophils [26], as well as in inflamed nasal tissue with allergic rhinitis [27]. However, we do not know about any report about Galectin-10 in nasal polyps of ASRD subjects. We know that the presence of nasal polyps in ASRD is the ultimate manifestation of a chronic inflamatory process with the presence of activated eosinophils and mast cells [28]; therefore, we could infer that galectin-10 could be helping to perpetuate the inflammatory process in this site. It is relevant to mention that galectin-10 or Charcot-Leyden crystals contain the enzyme lysolecithin acylhydrolase, which is one of several proteins involved in the eosinophil's immune functions [27]. Unfortunately, this enzyme also has the ability to damage the respiratory epithelium and to increase the vascular permeability [29, 30], which permits us to support its role in the chronic inflammatory response [31].

In this work we studied the levels of galectin-10 in nasal lavage fluid (NLF), before and after nasal challenge with L-aspirin. At baseline, the results evidenced higher concentrations of galectin-10 in NLF from patients with ASRD than in that from ATA and healthy subjects. After the challenge with L-aspirin, the levels of galectin-10 in the NLF behaved similarly; however, these differences were not significant between ASRD and ATA patients. We suggest that, after L-aspirin nasal challenge, the eosinophils migrate to the inflammation site in the nose, where they are activated and acquire the capacity to secrete their granule contents, increasing the levels of galectin-10 mainly in the ASRD group. In addition, we recently observed an increase in the concentration of eosinophil cationic protein (ECP) in NFL of aspirin-sensitive subjects versus healthy controls after nasal challenge with L-ASA [32], which confirms the release of mediators derived of eosinophils in the nasal fluid. 

Previously, Ghafouri and cols, in a proteomic study [22], reported the presence of galectin-10 in nasal lavage fluid (NLF) from patients with seasonal allergic rhinitis. This fact supports the possibility that galectin-10 plays a role in the exacerbation of the inflammatory response, as well as its potential utility in the correct diagnosis of ASRD. However, in spite of this finding, the role(s) of this galectin in the eosinophil, basophil, or associated inflammatory processes still needs to be established. Nevertheless, the results obtained in this work suggest the role of galectin-10 in the early inflammatory response in ASRD patients, which could be a useful factor for the accurate diagnosis of the disease. 

## Figures and Tables

**Table 1:** Clinical characteristics of the study group.

	HC	ATA	ASRD
Subjects (*n*)	15	10	15
AGE (years)	23 (21–34)	39 (14–62)	47 (28–74)
Gender M : F	8 : 7	4 : 6	2 : 13
Atopic (%)	0	30	47
Blood Eosinophiis (×10^3^/mm^3^)	0.17 ± 0.16	0.52 ± 0.26	0.50 ± 0.42*
IgE (IU/mL)	76.8 ± 65.4	229.5 ± 438.2	267.8 ± 320.7
Lisil-aspirin challenge (+)	0	0	15
Methacholine PC20 (mg/mL)	>32	3.0 ± 2	2.5 ± 1
Mean basal FEV (% predicted)	107 ± 6	103 ± 22	97 ± 12

HC: Healthy Controls.

ATA: Aspirin-Tolerant Asthmatics.

ASRD: Aspirin-Sensitive Respiratory Disease patients.

FEV: Forced-expiratory-volume.

*P > 0.05 ASRD versus ATA.
